# MO degradation by Ag–Ag_2_O/g-C_3_N_4_ composites under visible-light irradation

**DOI:** 10.1186/s40064-016-1805-5

**Published:** 2016-03-24

**Authors:** Xin Wang, Jia Yan, Haiyan Ji, Zhigang Chen, Yuanguo Xu, Liying Huang, Qi Zhang, Yanhua Song, Hui Xu, Huaming Li

**Affiliations:** School of Chemistry and Chemical Engineering, Institute for Energy Research, Jiangsu University, Zhenjiang, 212013 People’s Republic of China; Hainan Provincial Key Lab of Fine Chemistry, Hainan University, Haikou, 570228 Hainan People’s Republic of China; School of Environmental and Chemical Engineering, Jiangsu University of Science and Technology, Zhenjiang, 212003 People’s Republic of China

**Keywords:** Ag–Ag_2_O, g-C_3_N_4_, MO, Photocatalytic

## Abstract

The paper demonstrated the synthesis of Ag–Ag_2_O/g-C_3_N_4_ nanoparticles via a simple liquid phase synthesis path and a facile calcination method. The synthesized Ag–Ag_2_O/g-C_3_N_4_ composites were well characterized by various analytical techniques, such as X-ray diffraction, Fourier transform infrared (FT-IR), X-ray photoemission spectroscopy, transmission electron microscopy, scanning electron microscopy, high resolution transmission electron microscopy, the UV–Vis diffuse-reflectance spectra and transient photocurrent. From the structure and surface characterization, it indicated that Ag–Ag_2_O/g-C_3_N_4_ composites were formed by an effective covering of g-C_3_N_4_ with Ag–Ag_2_O. The results revealed that the 50 wt% nanoparticle had a great effection on the degradation of the methyl orange (MO), which was almost 7.5 times as high as that of g-C_3_N_4_. Based on the experimental results, the possible photocatalytic mechanism with photogenerated holes as the main active species was presented.

**Electronic supplementary material:**

The online version of this article (doi:10.1186/s40064-016-1805-5) contains supplementary material, which is available to authorized users.

## Background

With the development of the society, the environmental pollution has become one of the important problems which aroused more and more focus. It is well known that the TiO_2_ has been proved to be the most distinguished and widely used in the photocatalytic degradation of dyes (Liu et al. 2008; Chang et al. 2014) and H_2_ production (Cho et al. 2011; Park et al. 2006; Yang et al. 2009). However, with the increasing demands of the photocatalytic materials searching for more semiconductor photocatalysts is becoming more urgent. Thus, the mental and non-mental composites with g-C_3_N_4_ have attracted more attention (Peng et al. 2013; Zong et al. 2013).

As a good photocatalyst, ​Graphitic carbon nitride (g-C_3_N_4_) has been widely investigated since the discovery of its excellent properties by Liu and Cohen (1989). To date, it exhibits catalytic activity for extensive reactions, such as water splitting, oxidation reaction, dye photodegradation, nitric oxide (NO) decomposition and so on (Huang et al. 2013; Vignesh and Kang 2015; Chen et al. 2015; Yu et al. 2015; Dong et al. 2015; Chang et al. 2013; Su et al. 2010). This new material also possesses the good capabilities such as environmental friendly, stable, low cost and efficient. The reason why the g-C_3_N_4_ has a good photocatalytic activity is that the g-C_3_N_4_ possesses special optical characteristics and outstanding chemical stability. But, even with those merits, the g-C_3_N_4_ still has some disadvantages which show the limited photocatalytic property, such as the poor dispersion, easy agglomeration, recycling difficulties and so on. Yet combined with other materials such as the g-C_3_N_4_/MoO_3_ (Huang et al. 2013), g-C_3_N_4_/Ni(dmgH)_2_ (Cao et al. 2014), g-C_3_N_4_/Bi_2_O_2_CO_3_ (Tian et al. 2014), g-C_3_N_4_/Ag_3_PO_4_ (Xiu et al. 2014) and so on could enhance the catalytic activity of g-C_3_N_4_.

For example, in recent years, a g-C_3_N_4_ was modified with a composite semiconductor could possess the performance of water splitting and remove organic pollutants, which were reported by Wang et al. (2009) and Zhao et al. (2012). Wang and Zhang (2012) reported a g-C_3_N_4_–TiO_2_ pohotocatalyst fabricated by a simple impregnation method which has good activities for the H_2_ production. In fact, the approach indicates a synergetic effect of the impregnation preparation which provides a better junction between g-C_3_N_4_ and TiO_2_. It can be seen that the composites may have better photoactivities. However, not only can TiO_2_ doped possess the properties of degrading the pollutants, but also other mental and non-mental materials doped could have good activities. As we all know, the Ag-based materials have good photocatalytic activity. Thus, enormous efforts have been made to study more photocatalysts which needed Ag-based materials modification, such as Ag/C_3_N_4_ (Li et al. 2015), Ag/AgVO_3_/g-C_3_N_4_ (Zhao et al. 2015), Ag/AgCl/g-C_3_N_4_ (Yao et al. 2014), Ag–AgBr/g-C_3_N_4_ (Li et al. 2014) and so on.

In this paper, the Ag–Ag_2_O/g-C_3_N_4_ composites were successfully fabricated via a simple liquid phase synthesis path and a facile calcination method. The approach is different from the paper that has been reported by Xu et al. (2013) and Ren et al. (2014). The preparation of Ag–Ag_2_O can be described as following (Yu et al. 2014):

1 $$2{\text{AgNO}}_{3} + {\text{Na}}_{2} {\text{CO}}_{3} \to {\text{Ag}}_{2} {\text{CO}}_{3} \downarrow + \,2{\text{NaNO}}_{3}$$

2 $${\text{Ag}}_{2} {\text{CO}}_{3} \to {\text{Ag}}_{2} {\text{O}} + {\text{CO}}_{2} \uparrow$$

3 $$2{\text{Ag}}_{2} {\text{O}} \to 4{\text{Ag}} + {\text{O}}_{2} \uparrow$$

Simultaneously, Ag_2_O nanoparticles were partially reduced to Ag^0^ as it was calcined  at 220 °C for 90 min to prepare the desired Ag–Ag_2_O photocatalysts. This method is also used the same as the preparation of Ag–Ag_2_O/g-C_3_N_4_ nanocomposites. Then the intimate contacted interfaces between the Ag–Ag_2_O and g-C_3_N_4_ were also developed. In addition, prepared g-C_3_N_4_ via Ag–Ag_2_O doping has been proved to control the migration photon-generated carriers, so that the electrons and holes could be separated selectively at the edges, respectively. The mechanism of this report can explain phenomenon for it which indicates Ag–Ag_2_O has a great potential to be used as a stable and highly efficient photocatalyst to degrade the pollutants under the visible-light irradiation. MO, a representative of dyestuffs resistant to biodegradation, was selected as a model for the study. From our study, we find that the proportion of Ag–Ag_2_O loading on g-C_3_N_4_ surface has the most enhanced adsorption capacity and the best photocatalytic activity is 50 wt%. Therefore, both Ag and Ag_2_O maybe act as traps to capture photogenerated electrons which contribute to the separation of electron–hole pairs (Yu et al. 2005, 2012; Zhou et al. 2010; Subramanian et al. 2001; Xie et al. 2011). Based on the experimental results, a possible photocatalytic mechanism for the degradation of MO over Ag–Ag_2_O doped g-C_3_N_4_ nanosheets under visible-light irradiation was proposed.

## Experimental section

### Materials

All reagents in this work were AR grade and used without further purification.

### Preparation of g-C_3_N_4_

The g-C_3_N_4_ was synthesized by calcination method. In a typical process, 6 g dicyandiamide was put into three crucibles with three covers, sealed in a quartz tube partially backfilled with pure nitrogen, annealed at 350 °C for 2 h and annealed at 600 °C for 2 h again. Then the crucibles were cooled to room temperature.

### Preparation of Ag–Ag_2_O/g-C_3_N_4_ nanoparticles

The Ag–Ag_2_O/g-C_3_N_4_ was also synthesized via a simple liquid phase synthesis path and a facile calcination method. The method was as follow: 0.2 g of g-C_3_N_4_ was added into 20 ml of deionized water. Then they were magetic stirred for 5 min and sonicated for 15 min. Further, 0.2932 g of silver nitrate (AgNO_3_) was added into the solution and sonicated for 15 min. Next, 0.5 ml hydrated ammonia (NH_3_·H_2_O) was also added into the solution, which was still magetic stirred for 15 min. In addition, 0.1829 g of sodium carbonate (Na_2_CO_3_) was added drop by drop under stirring in 15 min. Moreover, the pH of the solution was adjusted to 7 and heated in water bath at 25 °C for 1 h. Next, the product was obtained by centrifugation, washed with ethanol and deionized water for several times and dried at 60 °C for 8 h. At last, the sample was annealed at 220 °C for 90 min. The 50 wt% Ag–Ag_2_O/g-C_3_N_4_ could be obtained. All the experiments were carried out at room temperature. The Ag–Ag_2_O/g-C_3_N_4_ composites with different mass ratios were synthesized using the same method through changing the amount of g-C_3_N_4_, AgNO_3_ and Na_2_CO_3_, such as 5, 10, 30 and 40 wt%, respectively.

### Characterization

The crystal phase of Ag–Ag_2_O, g-C_3_N_4_ and Ag–Ag_2_O/g-C_3_N_4_ powders were analyzed by X-ray diffraction (XRD) analysis using a Bruker D8 diffractometer with Cu-Kα radiation (λ = 1.5418 Å) in the 2θ range of 20°–80°. Scanning electron microscopy (SEM) image and transmission electron microscopy (TEM) micrographs were taken with a JEOL-JEM-2010 (JEOL, Japan) operating at 200 kV. High resolution transmission electron microscopy (HR-TEM) micrographs were taken with a FEI F20. Energy Dispersive spectrum (EDS) measurements were performed by a JEM-2100F electron microscope. The UV–Vis diffuse-reflectance spectra (DRS) of the samples were obtained on a UV–Vis spectrophotometer (UV-2450, Shimadzu Corporation, Japan). They were measured in solid state, and BaSO_4_ powder was used as the substrate. Fourier transform infrared (FT-IR) spectra of all the catalysts (KBr pellets) were recorded on Nicolet Model Nexus 470 IR equipment. X-ray photoemission spectroscopy (XPS) was measured on a PHI5300 with a monochromatic Mg Kα source to explore the elements on the surface. The photocurrents were measured with an electrochemical analyzer (CHI660B, CHI Shanghai, Inc.).

## Results and discussion

**Fig. 1:**
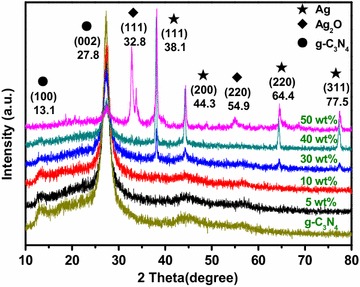
XRD patterns of Ag–Ag_2_O, g-C_3_N_4_ and Ag–Ag_2_O/g-C_3_N_4_ composite

The XRD patterns of the as-prepared Ag–Ag_2_O, g-C_3_N_4_ and Ag–Ag_2_O/g-C_3_N_4_ composites were shown in Fig. 1. All diffraction peaks could be indexed as “★” of Ag, “◆” of Ag_2_O, “●” of g-C_3_N_4_. The results indicated that the diffraction peak at 13.1° and 27.8° could be indexed as (100) and (002) diffraction planes (JCPDS 87-1526) (Wang et al. 2009). And the (100) diffraction peak is weakening with the decreasing content of g-C_3_N_4_. With the increasing Ag–Ag_2_O content, the diffraction peaks at 32.8° and 54.9° gradually appeared while the intensity increased, and the peaks were assigned to the (111) and (220) planes (JCPD 41-1104) (Wang et al. 2011) of Ag_2_O crystal, respectively. Four diffraction peaks at 32.8°, 44.3°, 64.4° and 77.5° in Ag were indexed to the (111), (200), (220) and (311) planes of Ag (JCPDS 04-0783) (Liu et al. 2015), respectively. As discussed above, the Ag–Ag_2_O/g-C_3_N_4_ nanocomposites were successfully prepared via a simple liquid phase synthesis path and a facile calcination method.

**Fig. 2:**
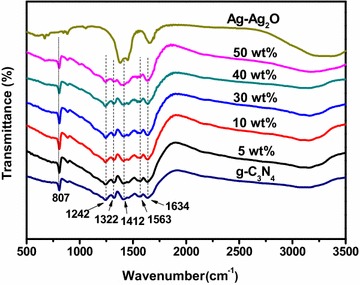
FT-IR of the as synthesized Ag–Ag_2_O, g-C_3_N_4_ and Ag–Ag_2_O/g-C_3_N_4_ composite

Figure 2 showed the FTIR spectra of the Ag–Ag_2_O, g-C_3_N_4_ and a series of Ag–Ag_2_O/g-C_3_N_4_ composite photocatalysts, respectively. The broad peak at 3000–3500 cm^−1^ was ascribed to the stretching vibration of N–H and that of O–H of the physically adsorbed water (Xu et al. 2013; Yan et al. 2014). In the case of g-C_3_N_4_, the strong band of 1200–1700 cm^−1^, with the characteristic peaks at 1242, 1322, 1412, 1563 and 1634 cm^−1^ were attributed to the typical stretching vibration of CN heterocycles (Xu et al. 2013; Yan et al. 2014). In addition, the peak at 807 cm^−1^ is associated with the breathing mode of triazine units (Min and Lu 2012; Lotsch and Schnick 2006). Moreover, for the Ag–Ag_2_O, the observed broad peak around 600 cm^−1^ belongs to Ag–O bond vibration (Xu et al. 2013). The FT-IR spectra of the Ag–Ag_2_O/g-C_3_N_4_ composites represented the spectra of both g-C_3_N_4_ and Ag–Ag_2_O. It should be noted that the intensity of the peak at 807 cm^−1^ decreased with the reduction of the g-C_3_N_4_ content.

**Fig. 3:**
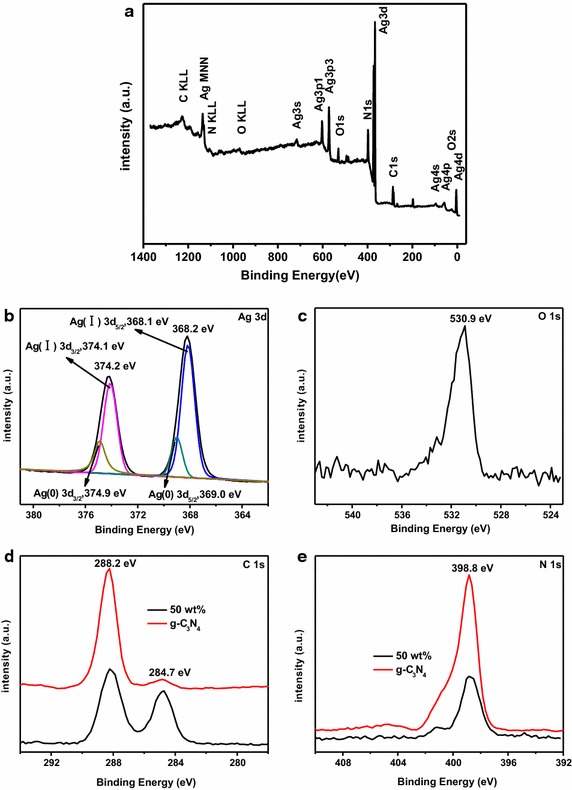
XPS spectra **a** profiles of survey, **b** Ag 3d, **c** O 1s, **d** C 1s and **e** N 1s

XPS was further made use of to analyze the chemical status and compositions of the 50 wt% Ag–Ag_2_O/g-C_3_N_4_ composite. Figure 3a showed the XPS analysis spectrum of the as-prepared composites, from which only Ag, O, C and N elements could be observed. In order to investigate the detailed chemical states of 50 wt% Ag–Ag_2_O/g-C_3_N_4_ nanoparticles, the peaks of Ag 3d, O 1s, C 1s and N 1s had been conducted and given in Fig. 3b–e. There were two peaks located at 374.2 and 368.2 eV could attach to the binding energies of Ag3d_5/2_ and Ag 3d_3/2_ (Melian et al. 2012), which belonged to Ag^+^ in Ag–Ag_2_O (Fig. 3b). Besides, the peak at 368.2 eV could be further divided into two bands of 368.1 eV and 369.0 eV for the binding energy of Ag(I) 3d_5/2_ and Ag(0) 3d_3/2_, respectively. And the peak at 374.2 eV could be also de-convoluted into two different peaks at 374.1 eV and 374.9 eV for Ag(I) 3d_5/2_ and Ag(0) 3d_3/2_, respectively. The peak centered at 530.9 eV could be attributed to the lattice oxygen atoms of Ag–Ag_2_O (Huang et al. 2013) (Fig. 3c). Figure 3d showed that the peaks located at 288.2 and 284.7 eV correspond to the sp^3^-bonded C in C–N of g-C_3_N_4_ and C–C coordination of the surface adventitious carbon (Li et al. 2013; Yan et al. 2012; Yan et al. 2010). Compared with the intensity of g-C_3_N_4_, the peak at 288.2 eV was strengthened and the peak at 284.7 eV was weakened. In the N 1s spectrum (Fig. 3e), the peak at 398.8 eV was assigned to C=N–C coordination (Wang et al. 2014), the intensity of which was stronger than that of g-C_3_N_4_. In the N 1s spectrum (Fig. 3e), the peak at 398.8 eV was assigned to C=N–C coordination (Wang et al. 2014). In the end, results from XRD, FT-IR and XPS indicated that the as-prepared samples contained Ag–Ag_2_O and g-C_3_N_4_.

**Fig. 4:**
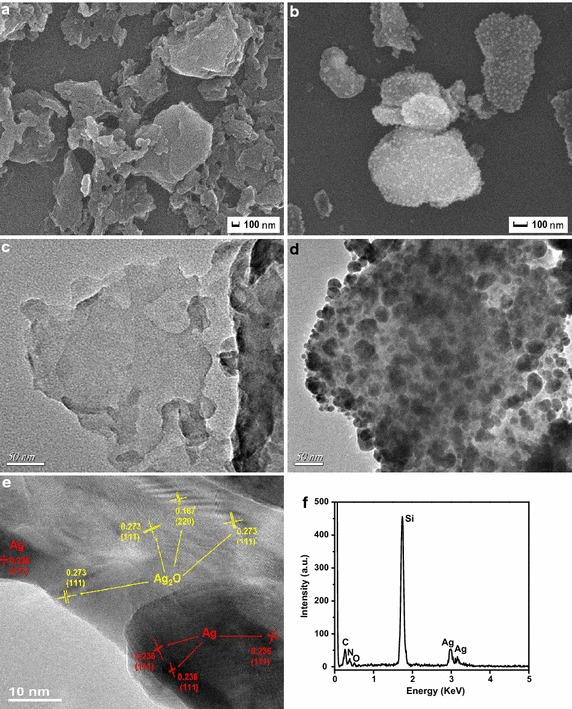
SEM morphologies of g-C_3_N_4_ (**a**), 50 wt% Ag–Ag_2_O/g-C_3_N_4_ (**b**), TEM morphologies of g-C_3_N_4_ (**c**), 50 wt% Ag–Ag_2_O/g-C_3_N_4_ (**d**), HR-TEM morphologies of 50 wt% Ag–Ag_2_O/g-C_3_N_4_ (**e**) and EDS of the 50 wt% Ag–Ag_2_O/g-C_3_N_4_ composite (**f**)

The morphological characterization of as-synthesized products was investigated by using SEM and TEM. SEM images were shown in Fig. 4a, b, which clearly depicted layer structure of g-C_3_N_4_ (Xu et al. 2013). From SEM images, it was obvious that these Ag–Ag_2_O nanoparticles were well dispersed on the surface of the g-C_3_N_4_. To further observe the combination of Ag–Ag_2_O and g-C_3_N_4_, EDS mapping images were shown in Additional file 1: Fig. S1, which indicated that Ag and O element were well distributed in the samples. TEM was used to investigate the morphology and microstructure of the sample. The TEM and HR-TEM images of 50 wt% Ag–Ag_2_O/g-C_3_N_4_ were shown in Fig. 4c–e. It can be seen that Ag–Ag_2_O particles were uniformly deposited on the surface of g-C_3_N_4_. The existence of heterojunction between Ag and Ag_2_O could be seen in the HR-TEM. Two different kinds of lattice fringes were clearly observed. The d = 0.236 of the first fringe matches the (111) crystallographic plane of Ag (Liu et al. 2015), and another of d = 0.273 and 0.167 nm are attached to the (111) and (220) crystallographic plane of Ag_2_O (Wang et al. 2011) respectively. What’s more, an integration interface between g-C_3_N_4_ and Ag–Ag_2_O is possibly formed, which was contributed to the transport of photoexcited carriers. At last, from the EDS, we could see that there were only Ag, O, C, N and Si elements, which consistent with the XRD in Fig. 4f. The corresponding EDS spectrum of the sample 50 wt% Ag–Ag_2_O/g-C_3_N_4_ confirmed that there were C, N, O, Si and Ag elements in the sample as shown in Fig. 4f. Also from the Additional file 1: Table S1, the actual data of the content of Ag–Ag_2_O in the sample were close to the theoretical data of that. Even though there were some differences between the theoretical data and the actual data, these might be due to the loss of g-C_3_N_4_ in the calcination process. In addition, the observed Si peaks in the above EDS spectrum arose from the silicon grids was used for SEM analysis.

**Fig. 5:**
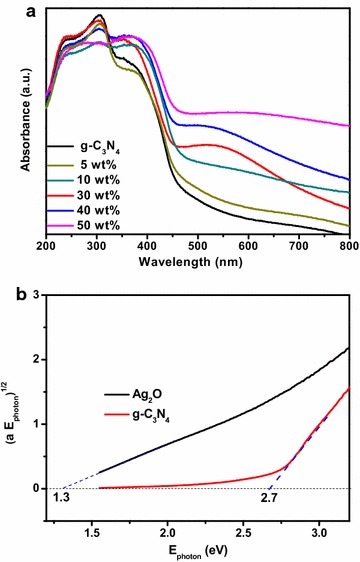
DRS (**a**), versus photon energy of the Ag–Ag_2_O/g-C_3_N_4_ treated for different proportions and plots of (αhυ)^1/2^ (**b**) versus photon energy of the Ag_2_O and g-C_3_N_4_

The DRS of Ag–Ag_2_O/g-C_3_N_4_, Ag–Ag_2_O and g-C_3_N_4_ were shown in Fig. 5. The absorption edges were varied by changing the amount of Ag–Ag_2_O. As shown in Fig. 5a, the g-C_3_N_4_ had the absorption edge of around 460 nm. When the ratio of Ag–Ag_2_O/g-C_3_N_4_ was increased from 5 to 50 wt%, the absorption edge of the composites shifted to the larger wavelength region and the composites exhibited stronger absorbance in the visible region due to the surface plasmon resonance (SPR) absorption of metal Ag nanocrystal. Compared with the 30 wt% and 50 wt% Ag–Ag_2_O/g-C_3_N_4_ composites, the 30 wt% Ag–Ag_2_O/g-C_3_N_4_ showed more obvious SPR than 50 wt% Ag–Ag_2_O/g-C_3_N_4_ which had more content of Ag–Ag_2_O attached to the surface of g-C_3_N_4_, that leaded to the absorption peak widen and then changed the SPR (Xu et al. 2011). The band gap values (E_g_) of Ag–Ag_2_O and g-C_3_N_4_ were calculated by plots of (αhυ)^1/2^ versus photon energy, which were shown in Fig. 5b. From the Fig. 5b, the band gap energy of g-C_3_N_4_ was 2.7 eV. At the same time, the band energy of Ag–Ag_2_O was 1.3 eV, which would be used in the possible mechanism at the end. To give a direct analysis, the potentials of the conduction band (CB) and valence band (VB) edges of g-C_3_N_4_ and Ag_2_O were evaluated by Mulliken electronegativity theory:

$${\text{E}}_{\text{CB}} = {\text{X}} - {\text{E}}_{\text{C}} - 1/2{\text{E}}_{\text{g}}$$

$${\text{E}}_{\text{CB}} = {\text{E}}_{\text{VB}} - {\text{E}}_{\text{g}}$$

where X was the absolute electronegativity of the atom semiconductor [(X_Ag2O_ = 4.44 * 4.44 * 7.54)^1/3^ = 5.29], defined as the geometric mean of the absolute electronegativity of the constituent atoms, and expressed as the arithmetic mean of the atomic electro affinity and the first ionization energy; E_C_ was the energy of free electrons with the hydrogen scale (4.5 eV); E_g_ was the band gap of the semiconductor (Xu et al. 2013). Based on the band gap positions, the CB and VB edge potentials of Ag_2_O were at +0.14 eV and +1.44 eV, respectively. The CB and VB edge potentials of g-C_3_N_4_ were at −1.13 eV and +1.57 eV, which were consistent with the previous literature, respectively (Xu et al. 2013).

**Fig. 6:**
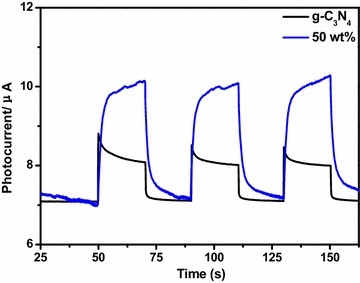
Transient photocurrent response for g-C_3_N_4_ and 50 wt% Ag–Ag_2_O/g-C_3_N_4_ composite

Commonly, a high value of the photocurrent demonstrates that the composite holds strong ability in generating and transferring the photoexcited charge carrier under irradiation. As shown in Fig. 6, the g-C_3_N_4_ and different ratios of Ag–Ag_2_O/g-C_3_N_4_ composite were characterized by transient photocurrent. The 50 wt% Ag–Ag_2_O/g-C_3_N_4_ had a higher photocurrent than g-C_3_N_4_, which indicates that Ag–Ag_2_O/g-C_3_N_4_ composite exhibits stronger ability than g-C_3_N_4_ in the separation of electron–hole pairs. While under visible-light irradiation, the pure g-C_3_N_4_ showed lower photocurrent response, because of its lower efficiency of the charge carriers’ separation. The results in Fig. 6 could well correspond to those from the MO degradation experiments which were shown as the following.

**Fig. 7:**
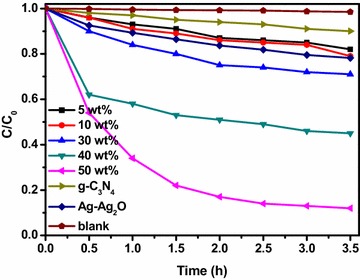
MO dye degradation curves of the g-C_3_N_4_ and Ag–Ag_2_O/g-C_3_N_4_ with different Ag–Ag_2_O modifying amount under visible light irradiation

Figure 7 showed the MO degradation curves of the photocatalysts of g-C_3_N_4_ and Ag–Ag_2_O/g-C_3_N_4_ with different Ag–Ag_2_O modifying amount under visible light irradiation. As shown in Fig. 7, the g-C_3_N_4_ showed poor activity, on which ~12 % of MO was decomposed after visible light irradiation for 3.5 h. After combining Ag–Ag_2_O with g-C_3_N_4_, the experiments clearly demonstrated that the Ag–Ag_2_O/g-C_3_N_4_ composite was determined as an efficient visible light photocatalyst, which was higher than the g-C_3_N_4_. Above all, the photoactivity of 50 wt% Ag–Ag_2_O/g-C_3_N_4_ composite was about 7.5 times higher compared to g-C_3_N_4_ and had the best photoactivity of all. The results may according to that there is a heterojunction between the Ag–Ag_2_O and g-C_3_N_4_, which can improve separation of electron–holes pairs and therefore enhance the photocatalytic activity of the g-C_3_N_4_.

**Fig. 8:**
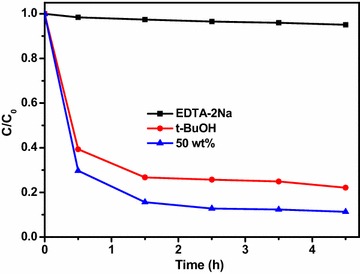
Effect of different scavengers on the degradation of methyl orange in the presence of 50 wt% Ag–Ag_2_O/g-C_3_N_4_ composites under visible light irradiation

Hydroxyl radicals and photogenerated holes are two main species for the oxidization of organic molecular in aqueous solution. In order to understand the photocatalysis profoundly, the effects of holes and hydroxyl radicals on the photocatalytic evaluation were investigated. As shown in Fig. 8, due to the tert-Butyl alcohol (TBA) could efficiently entrap the ·OH radicals, which was selected as ·OH scavenger. The change for the photodegradation of MO was small of the TBA, revealing that the hydroxyl radicals were not the main active species. However, after introducing EDTA-2Na as a hole scavenger, the photodegradation efficiency of MO over Ag–Ag_2_O/g-C_3_N_4_ greatly reduced from 95 to 11 % after irradiation for 4.5 h. These results indicated that the holes played an important role in the degradation of MO over Ag–Ag_2_O/g-C_3_N_4_.

**Fig. 9:**
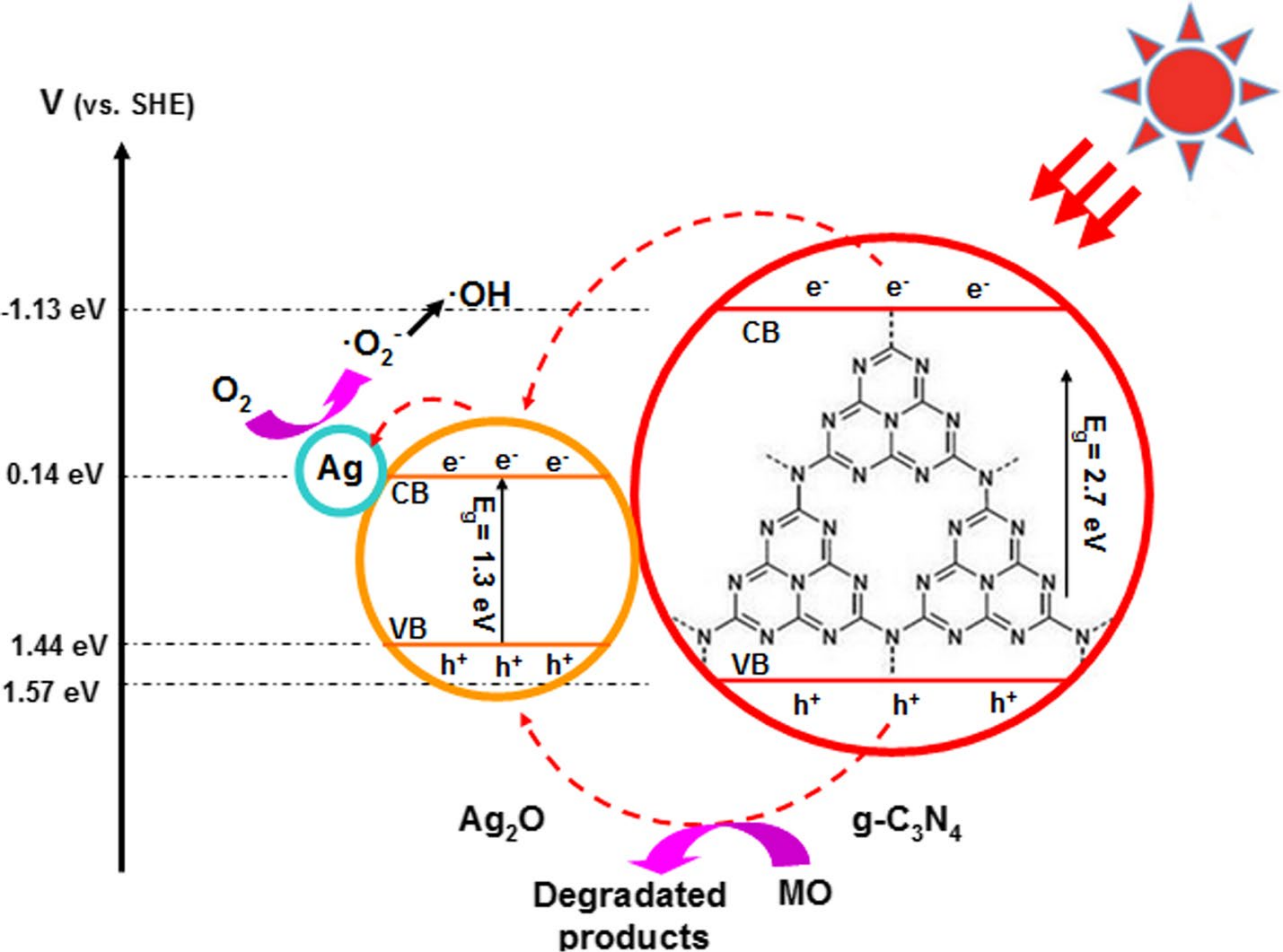
Proposed mechanism for the promotion of the photocatalytic MO degradation performance of Ag–Ag_2_O/g-C_3_N_4_ composites

The Fig. 9 showed the possible mechanism of photodegradation of MO over Ag–Ag_2_O/g-C_3_N_4_ photocatalyst under visible-light irradiation as follows. When under the visible-light exposure, both of the Ag_2_O and g-C_3_N_4_ generate valence band holes (h^+^) and conduction band electrons (e^−^). In order to give a direct analysis, the potentials of the conduction band (CB) and valence band (VB) edges of Ag_2_O and g-C_3_N_4_ were evaluated by Mulliken electronegativity theory (Xu et al. 2013). Due to the valence band potential of Ag_2_O was more negative than that of g-C_3_N_4_ and the conduction band potential of Ag_2_O was more positive than that of g-C_3_N_4_, the photoinduced holes on the valence band and the electrons on the conduction band of g-C_3_N_4_ could move to Ag_2_O.

In addition, the metallic Ag can further complete efficient electron migration process to efficiently inhibit the recombination of the photoexcited pairs (Xu et al. 2013). So it can be seen that even the VB and CB of g-C_3_N_4_ are higher than that of Ag_2_O, the Ag can be worked as the charge transmission bridge, which transfers the photogenerated electrons from the CB of Ag_2_O to Ag^0^ and then the photogenerated electrons were trapped by O_2_ to produce **·**O_2_^−^. At last, the **·**O_2_^−^ transformed into **·**OH. As a result, with the assistance of Ag–Ag_2_O, the Ag–Ag_2_O/g-C_3_N_4_ photocatalysts could effectively enhance the separation of photoexcited electron-hole pairs and reduced the recombination of electrons and holes. Thus, the Ag–Ag_2_O nanoparticles loaded on the surface of the g-C_3_N_4_ could form the heterojunction structure, which contributed to the promotion of the photocatalytic activity.

## Conclusion

In summary, we have demonstrated that Ag–Ag_2_O nanophases were active catalysts for degrading MO. The results revealed that the optimal activity of Ag–Ag_2_O/g-C_3_N_4_ is 7.5 times as high as that of g-C_3_N_4_ and even better than that of Ag–Ag_2_O. In this investigation the as-synthesized samples were characterized by a collection of techniques, such as XRD, SEM, TEM, HR-TEM, DRS, EDS, XPS and FT-IR. Based on structural analysis, we concluded that the Ag–Ag_2_O nanoparticles are dispersed on the surface of the g-C_3_N_4_. The modified g-C_3_N_4_ samples were robust and able to show better photocatalytic activities than Ag–Ag_2_O and g-C_3_N_4_. In addition, the photocatalysis mechanism was also investigated by entrapping active species. These results indicated that the holes played important roles in the degradation of MO over sample Ag–Ag_2_O/g-C_3_N_4_.

## Additional file

10.1186/s40064-016-1805-5 MO degradation by Ag-Ag_2_O/g-C_3_N_4_ composites under visible-light irradation.
