# Supplementary material for: MO degradation by Ag–Ag2O/g-C3N4 composites under visible-light irradation
Source: Springerplus. 2016 Mar 24;5:369. doi: 10.1186/s40064-016-1805-5 (PMC4805675; doi:10.1186/s40064-016-1805-5)
Supplement: Supplementary file 1 — 10.1186/s40064-016-1805-5 MO degradation by Ag-Ag2O/g-C3N4 composites under visible-light irradation. [file 40064_2016_1805_MOESM1_ESM.doc]

**MO Degradation by Ag-Ag_2_O/g-C_3_N_4_ Composites Under Visible-light Irradation**

**Xin Wang^a^, Jia Yan^a^, Haiyan Ji^a^, Zhigang Chen^a^, Yuanguo Xu^a^, Liying Huang^a^, Qi Zhang^b^, Yanhua Song^c^, Hui Xu^a,*^, Huaming Li**^a, *^

^a^School of Chemistry and Chemical Engineering, Institute for Energy Research, Jiangsu University, Zhenjiang 212013, P. R. China

^b^Hainan Provincial Key Lab of Fine Chemistry, Hainan University, Haikou, Hainan 570228, P.R. China

^c^School of Environmental and Chemical, Engineering, Jiangsu University of Science and Technology, Zhenjiang 212003, P. R. China

***Corresponding author:** Tel.:+86-0511-88791108; Fax: +86-0511-88791108;

E-mail address: xh@ujs.edu.cn, lihm@ujs.edu.cn

Table S1 The actual content of Ag and Ag-Ag_2_O in different proportions of Ag-Ag_2_O/g-C_3_N_4_ by EDS.

| Theoretical content (Ag-Ag_2_O wt%) | 5 | 10 | 30 | 40 | 50 |
| --- | --- | --- | --- | --- | --- |
| Experimental  (Ag wt%) | 1.65 | 6.39 | 21.94 | 34.96 | 45.57 |
| Experimental  (Ag-Ag_2_O, wt%) | 8.47 | 13.42 | 29.6 | 41.61 | 56.38 |

**
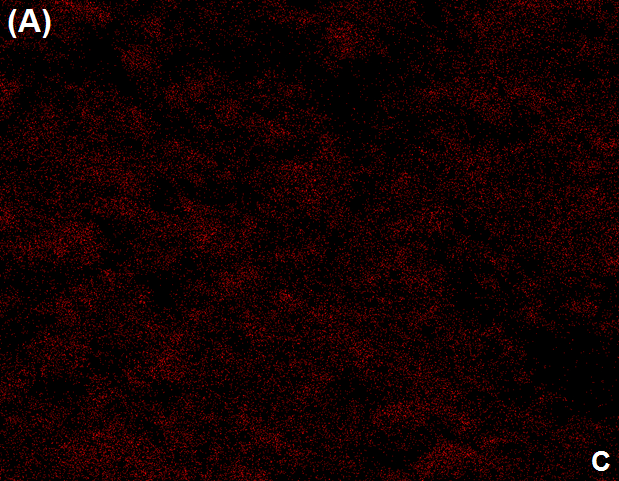

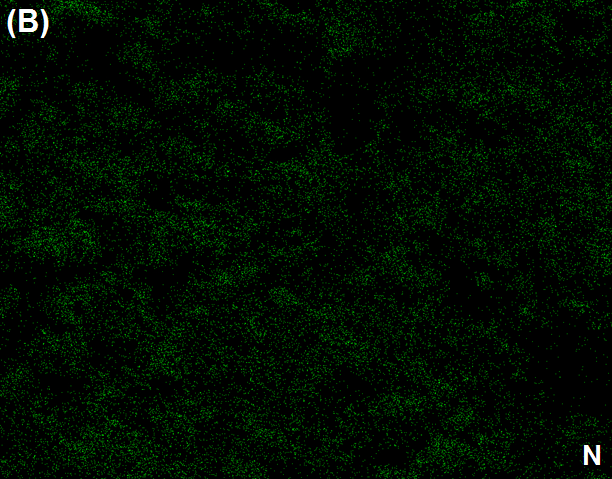

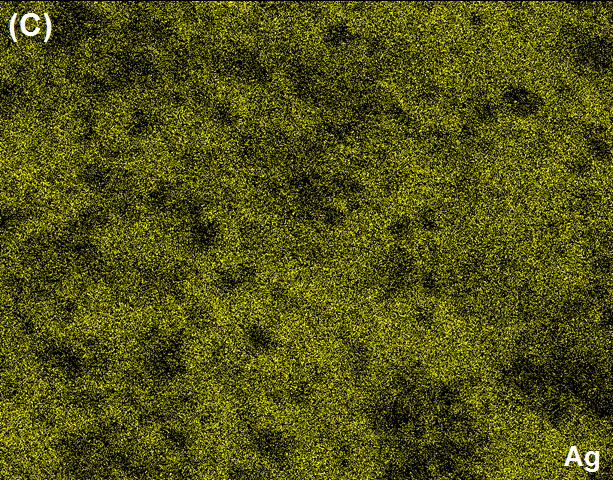

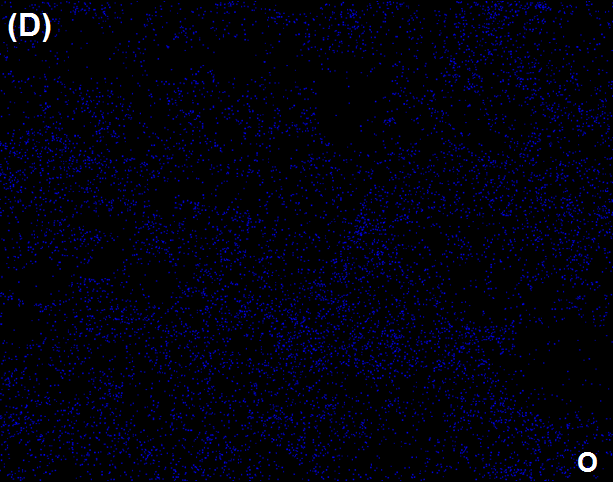
**

Fig. S1. High-angle annular dark-field (HAADF) scanning transmission electron microscopy (STEM) of the 50 wt% Ag-Ag_2_O/g-C_3_N_4_, the spatially resolved C (A), N (B), Ag (C) and O (D).
